# Commentary on: Factors influencing foot care behaviour among patients with diabetes: An integrative literature review by Woo MWJ, CUI J (2023)

**DOI:** 10.1111/jocn.17391

**Published:** 2024-08-16

**Authors:** Lara Delbene, Milko Zanini, Gianluca Catania, Giuseppe Aleo, Annamaria Bagnasco

**Affiliations:** ^1^ Department of Health Sciences University of Genoa Genoa Italy; ^2^ Faculty of Nursing & Midwifery Royal College of Surgeons in Ireland Dublin Ireland

**Keywords:** Barrows Cards, diabetic foot care behaviours, education, educational intervention

## Abstract

The review by Woo et al. reports on factors influencing behaviour in the care of the diabetic foot, wich are common in diabetic patients and have a high risk of infection and amputation. To improve patient's knowledge and education on foot care, this commentary proposes the Barrows cards as an innovative user‐friendly educational method. Conclusion and implications for profession and patient care: adapting these cards to adult diabetic patients could prevent future complications, improving quality of life and reduce the risks associated with diabetes. No patient or public contribution.

We aim to comment the integrative review by Woo et al., entitled ‘Factors influencing foot care behaviour among patients with diabetes: An integrative literature review’.

This study is particularly relevant as it identifies key factors influencing behaviour in diabetic foot care. The results obtained may serve as a basis for future therapeutic and educational interventions aimed at improving this crucial aspect for the quality of life and health of diabetic patients.

The authors of the review pointed out that diabetic foot care behaviour is often inadequate among diabetic patients. Approximately one‐third of the global diabetic population develops diabetic foot diseases, and half of the poorly treated cases tend to evolve into foot infections (Armstrong et al., 2020). These infections, if not properly treated, can lead to lower limb amputations and are the leading cause of hospitalization among diabetic patients (Dewi & Hinchliffe, 2020). Moreover, the healthcare costs associated with treating diabetic foot diseases place a significant burden on both patients and public healthcare systems (Rinkel et al., 2017).

As such, the review identified the main factors influencing diabetic foot care behaviour, including demographic characteristics, beliefs and perceptions of the disease, knowledge of foot care and foot care education.

Barrows Cards offer an innovative educational strategy that could effectively integrate at least the last two key influencing factors (i.e., knowledge of foot care and foot care education). Barrows Cards, also known as the Portable Patient Problem Pack or P4 System, are an educational method to test decision‐making skills and critical thinking in medical students (Barrows & Tamblyn, 1977). Using a deck of at least 15 cards, students tackle complex problems independently, choosing and sequencing appropriate actions. This method simulates real clinical situations, allowing students to make mistakes and improve their problem‐solving skills. In the mixed‐methods study by Bagnasco et al. (2016), the cards were adapted as an educational intervention to improve self‐management and adherence to immunosuppressive treatment in a group of adolescents with blood cancer to prevent serious life‐threatening complications requiring bone marrow replacement surgery.

In this study, the educational intervention consisted of presenting adolescent patients with an initial card called the “situational card,” which described a problem. Next, they were given a deck of at least 15 cards, each illustrating a different behaviour. Patients had to choose the card showing the correct behaviour to solve the problem presented in the situational card. The front side of each card described a behaviour, which could be correct, partially correct or wrong, while on the back of the card it shows whether the chosen behaviour was correct, partially correct or wrong and why.

Adapting this educational intervention to include adult diabetic patients, who do not yet have diabetic foot complications, could prove useful. A preventive approach would avoid potential future complications while improving foot healthcare and maintenance behaviours. Early and targeted education could thus contribute significantly to a better quality of life and a reduction in the risks associated with diabetes.

These cards can present realistic situations related to diabetic foot management, illustrating both incorrect and correct behaviours. Through a simple and customizable approach, Barrows Cards can specifically address, for instance, misconceptions, providing clear and accurate information to improve knowledge and promote preventive behaviours.

In conclusion, the adoption of Barrows Cards as an integral part of diabetic foot care education offers significant potential to enhance education and adherence to preventive practices. By integrating the key influencing factors, these cards offer a comprehensive and personalized approach that can significantly contribute to reducing the risk of severe complications associated with diabetic foot and improving the overall clinical outcome of patients with diabetes mellitus.

## AUTHOR CONTRIBUTIONS

LD: Made substantial contributions to conception and design, or acquisition of data or analysis and interpretation of data; LD: Involved in drafting the manuscript or revising it critically for important intellectual content; LD: Given final approval of the version to be published. Each author should have participated sufficiently in the work to take public responsibility for appropriate portions of the content; LD, MZ, GC, GA and AB: Agreed to be accountable for all aspects of the work in ensuring that questions related to the accuracy or integrity of any part of the work are appropriately investigated and resolved.

## FUNDING INFORMATION

This research received no specific grant from any funding agency in the public, commercial or not‐for‐profit sectors.

## CONFLICT OF INTEREST STATEMENT

Authors declare that they have no conflicts of interest with respect to the research, authorship and/or publication of this article.

## ETHICS STATEMENT

No ethics approval was required for this commentary.

## Data Availability

Not applicable.
